# Marine Tar Residues: a Review

**DOI:** 10.1007/s11270-015-2298-5

**Published:** 2015-02-25

**Authors:** April M. Warnock, Scott C. Hagen, Davina L. Passeri

**Affiliations:** 1 Communications, Radar and Sensing Group, SRI International, 2100 Commonwealth Boulevard, Ann Arbor, MI 48105 USA; 2 Department of Civil, Environmental, and Construction Engineering, University of Central Florida, 12800 Pegasus Blvd, Suite 211, Orlando, FL 32816-2450 USA; 3 Present Address: Department of Civil and Environmental Engineering/Center for Computation and Technology, Louisiana State University, 3418 Patrick F. Taylor, Baton Rouge, LA 70803 USA

**Keywords:** Marine tar, Tar balls, Tar mats, Oil spills

## Abstract

Marine tar residues originate from natural and anthropogenic oil releases into the ocean environment and are formed after liquid petroleum is transformed by weathering, sedimentation, and other processes. Tar balls, tar mats, and tar patties are common examples of marine tar residues and can range in size from millimeters in diameter (tar balls) to several meters in length and width (tar mats). These residues can remain in the ocean environment indefinitely, decomposing or becoming buried in the sea floor. However, in many cases, they are transported ashore via currents and waves where they pose a concern to coastal recreation activities, the seafood industry and may have negative effects on wildlife. This review summarizes the current state of knowledge on marine tar residue formation, transport, degradation, and distribution. Methods of detection and removal of marine tar residues and their possible ecological effects are discussed, in addition to topics of marine tar research that warrant further investigation. Emphasis is placed on benthic tar residues, with a focus on the remnants of the Deepwater Horizon oil spill in particular, which are still affecting the northern Gulf of Mexico shores years after the leaking submarine well was capped.

## Introduction

The term “marine tar residue” used herein describes several types of weathered oil conglomerations that originate from marine oil pollution and can be found in varying quantities on beaches, the open ocean surface, and the seafloor. This paper provides a comprehensive literature review of marine tar-related research from the 1970s to the present, including an overview of classification and terminology, quantitative/qualitative descriptions of marine tars, a review of papers that have been published on tar distribution and prevalence, chemical composition and tracing, transport mechanisms, formation and degradation, as well as human and ecological effects. Particular focus is given to benthic tar residues in light of their pervasiveness in the Gulf of Mexico following the Deepwater Horizon oil. Overall, the goal of this paper is to provide a unified and comprehensive resource on the topic of marine tar residues as well as to elucidate areas in need of further study.

### Tar Residue Types and Formation Mechanisms

Marine tar residues result from natural and anthropogenic oil pollution. Anthropogenic sources of marine oil include emissions from oil exploration, consumption, and transportation, while naturally occurring petroleum seeps on the seafloor provide a chronic source of oil pollution (NAS 2003). The contributions of these various sources are difficult to quantify and fluctuate in response to petroleum industry productivity as well as frequency of tanker accidents and well blowouts. Further confusing the determination of contributions is the interrelation between petroleum industry activity and the rate of discharge from natural seeps (Kvenvolden and Cooper 2003), as well as the inherent variability of seep emissions over time (Leifer et al. 2004). Recent estimates have determined that a little over half of the global marine tar load comes from anthropogenic sources, with the remainder coming from natural oil seeps (NAS 2003). Several studies from decades past have tied the prevalence of marine tars to nearby tanker routes (e.g., NAS 1975; Wong et al. 1976; Atwood et al. 1987), likely the result of “operational discharges of oil,” which include deliberate, routine releases of oil and tar from ballast tanks and from the washing out of tanker storage compartments (Ehrhardt and Blumer 1972; NAS 2003). Stricter regulations in recent decades (e.g., MARPOL 73/78) have reduced the marine oil pollution from routine tanker operations (Day and Shaw 1987; Golik et al. 1988; NAS 2003).

**Fig. 1:**
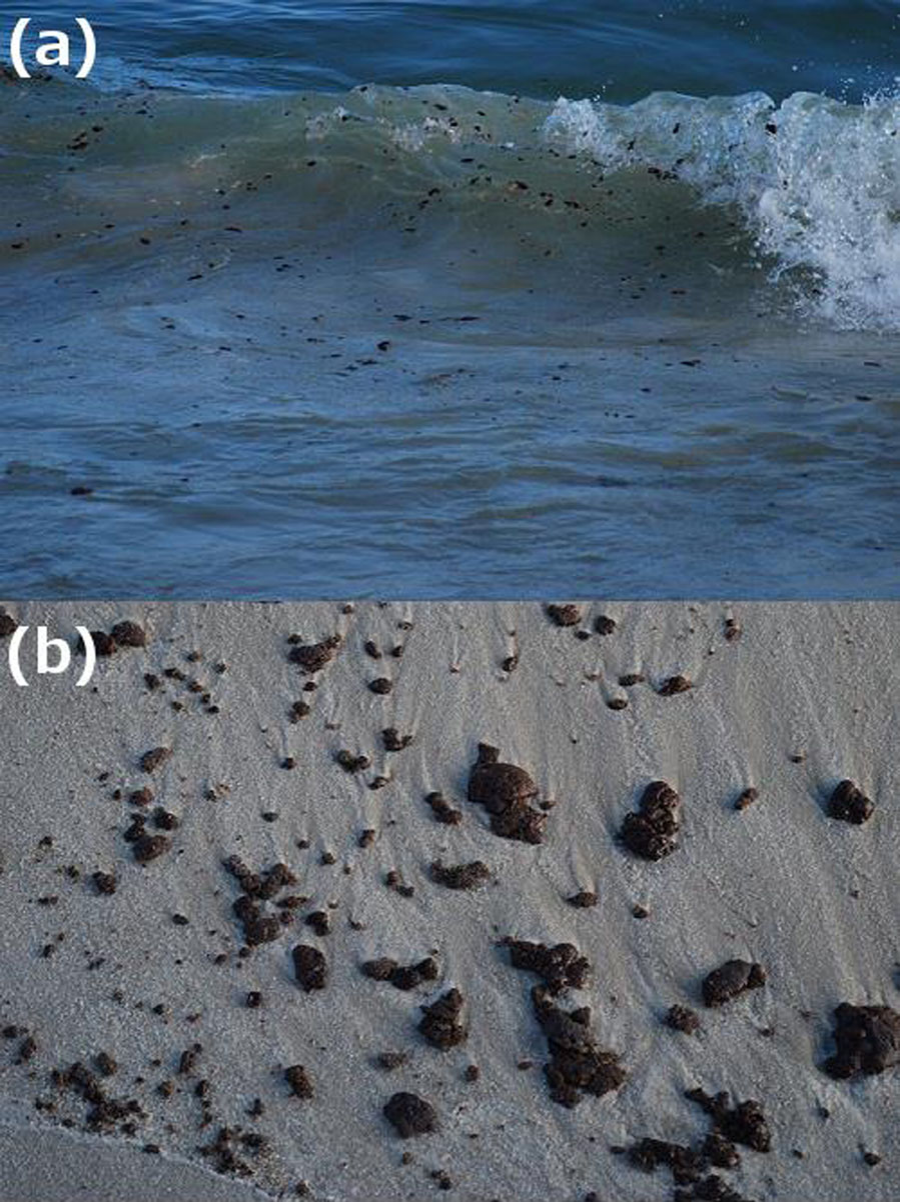
**a** Pelagic (*floating*) tar balls from the Deepwater Horizon spill; **b** beached tar balls (http://www.opednews.com/articles/MAN-VS-OIL-by-Emily-McDaniel-100614-242.html)

**Fig. 2:**
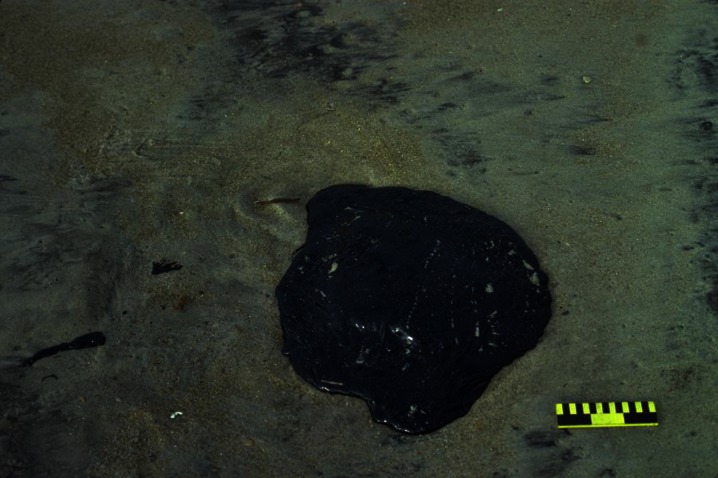
Beached tar patty, approximately 40 cm in diameter. Photo credit: Miles O. Hayes and Jacqueline Michel of Research Planning, Inc. (http://response.restoration.noaa.gov/surface-oiling-descriptors-type)

**Fig. 3:**
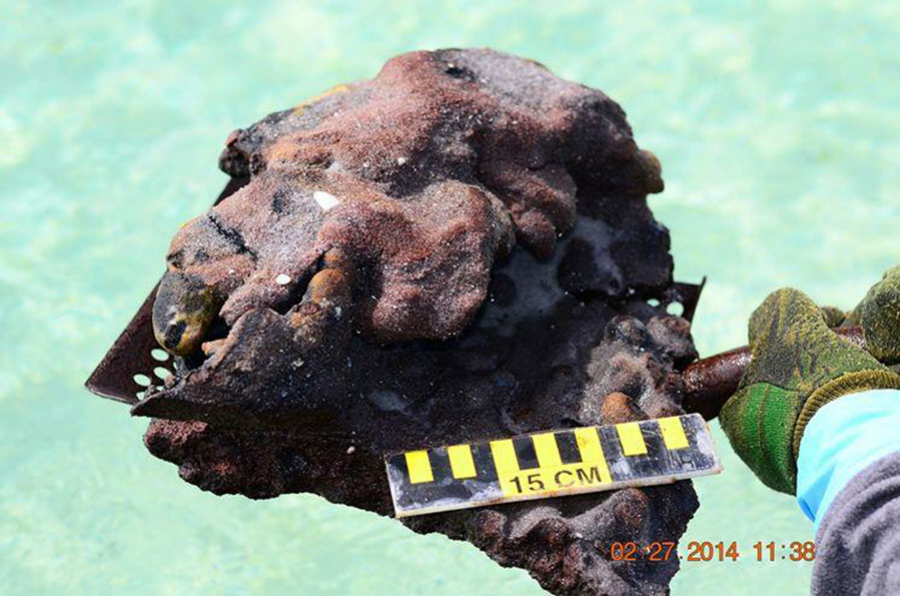
Tar mat piece recovered from Pensacola Beach in February, 2014 (http://www.tampabay.com/news/environment/water/four-years-after-oil-spill-1250-pound-tar-mat-washes-ashore-in-florida/2167938)

Marine tar residues vary considerably in color, shape, size, chemical makeup, and aroma. A number of distinguishing terms have been used to characterize these various types of tar. Commonly, descriptions are based on size. The term tar ball is used to describe a discrete, roughly spherical accumulation of weathered oil generally less than 10 cm in diameter (Fig. 1). Discrete tar aggregates that are larger than 10 cm in diameter are referred to as tar patties (Fig. 2) (Wang and Roberts 2013; California Department of Fish and Wildlife 2013). Large, thick accumulations of oil residues that are partially or completely submerged by water are referred to as tar mats (Fig. 3). Marine tars can also be categorized as pelagic or benthic. Pelagic tars are observed floating or shallowly submerged on the sea surface (Fig. 1a), while benthic tar residues reside on the seafloor. Both pelagic and benthic tar residues can be transported to the shore by waves and currents, resulting in beached or “stranded” tar (Fig. 1b) (Bernabeu et al. 2013; Iliffe and Knap 1979; Butler et al. 1998).

**Fig. 4:**
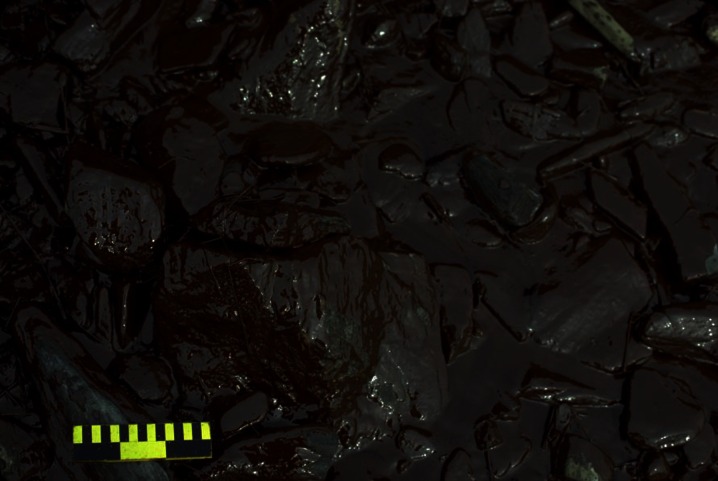
“Chocolate mousse.” Photo credit: Miles O. Hayes and Jacqueline Michel of Research Planning, Inc.

The formation of marine tar residues from liquid oil is not a fully understood process, and several theories have been put forth (see Goodman 2003). With respect to pelagic tars in particular, perhaps the most widely accepted explanation is what will be referred to herein as the surface-weathering theory. This theory assumes that tar residues originate directly from the weathering of oils at the sea surface. Weathering, a combination of processes, including spreading, evaporation, dissolution, biodegradation, emulsification, sedimentation, dispersion, and oxidation, leaves behind oil components that are heavier and more viscous (NAS 2003); with emulsification, a water-in-oil mixture forms that is referred to as “chocolate mousse” or “mousse” (Fig. 4) (Payne 1982; Thingstad and Pengerud 1983; NAS 2003; Fingas and Fieldhouse 2009). This weathered emulsion then breaks into pieces, forming pelagic tar balls or patties.

Pelagic tar balls may become benthic after being subjected to processes that increase their specific gravity. These processes include continued weathering at sea or ambient temperature changes (Iliffe and Knap 1979; Balkas et al. 1982), colonization of organisms such as barnacles and isopods (Horn et al. 1970; Okera 1974), or sediment accumulation after having been tossed ashore and picking up sediments on the beach. The beached tar balls may then be washed back to sea, where they are now too heavy to float (Golik 1982). Although tar residues that form directly from surface weathering can become encrusted with sediments or organisms, the inner core can remain soft and gooey and may even reliquify at high temperatures once beached (Hooper 1981; Georges and Oostdam 1983; NOAA 2010).

While the surface-weathering theory is sufficient to explain the formation of many marine tar residues, some residues arise primarily from the mixing of oils with sediments instead of strictly surface weathering. Included in this category of “sedimented” tar residues are tar balls that form via erosion of oiled sands on polluted beaches (Michel et al. 1993; Hayes et al. 1993) and the tar aggregates that may result from spills of heavy or viscous oils, properties that increase the likelihood of the oil sinking, and the entrainment of sand and shell particles (Michel and Galt 1995). Considering that sand is primarily composed of quartz, with a specific gravity of 2.65, oil that has mixed with as little as 2 % sand by weight will lose its buoyancy and sink (National Research Council 1999). Entrainment of sediments is more likely to occur in high-energy (i.e., shallow, nearshore) environments. Therefore, floating oil that is transported to a shallower region can form large agglomerations of sunken oil, sand, and shell, which are deposited in depressions in the sea beds (National Research Council 1999; OSAT-3 2013). Pieces of these highly sedimented sand/oil agglomerates can break off into benthic tar balls (OSAT-2 2011; OSAT-3 2013). Due to the large percentage of sediments directly incorporated in this category of tar residues, they tend to be less “tarry” in nature (e.g., softer and more fragile) than tar residues resulting directly from surface weathering. The Deepwater Horizon (DWH) oil spill, which released an estimated 700,000 m^3^ of oil into the Gulf of Mexico from April 20 to July 15, 2010 (Crone and Tolstoy 2010), resulted in a large number of these sedimented type oil residues in the intertidal and subtidal zones of the affected coastal regions (Alabama, Louisiana, Mississippi, and Florida), as will be discussed further below.

The possibility of tar residues forming due to natural seeps must also be mentioned. Fischer and Stevenson (1973) proposed the idea that oil leaking from benthic seeps off the coast of California undergoes rapid separation into heavier and lighter fractions. The heavier fraction is subject to flocculation and sedimentation, resulting in its sinking and accumulating in troughs in the ocean basin. They bolstered their theory with evidence from sediment core observations in the region that showed layers of tar sandwiched between other sediments.

The time required to form marine tar residues from liquid oil has not been clearly established. Reports have indicated that tar mats and patties can form in a matter of days after a spill (Clark et al. 1997) while as discussed below, laboratory created tar balls may take months to form. In the case of the DWH spill, tar balls conclusively linked to DWH oil were recorded washing ashore on beaches from Pensacola, FL, to Galveston, TX, less than 2 months after the spill commenced (Jonsson 2010; Woodham 2010); however, it is not known how much of this lag time is attributable to the transport of the tar balls from offshore to the coasts.

### Laboratory Experiments

The few laboratory studies that have been carried out to simulate marine tar formation have attempted to replicate pelagic tar balls formed by some combination of surface-weathering processes. MacGregor and McLean (1977) sought to reproduce conditions under which tar balls form in cold marine water. They used a ratio of 1 gal crude oil to 100 gal manufactured seawater in a tank that was aerated with bubbles, stirred mechanically, and exposed to a sunlamp. They also included control experiments in which a single weathering process was isolated in order to investigate the effects of evaporation and oxidation. Under the baseline conditions, tar balls began to form after about 2 weeks. Evaporation was found to decrease after about 400 h of radiation exposure, correlating to approximately 85 % of remaining oil volume. The increase in specific gravity of the tar balls was determined to be due to the emulsification process. Without the incorporation of seawater, the increase in specific gravity was estimated to be only 0.85 to 0.93. With seawater, the specific gravity leveled out at around 1.00. Based on their observation that there were no discernible differences between the outer skin and inner portion of the tar balls, the authors concluded that the formation of tar balls is not dependent on weathering processes. However, this experiment could not account for all of the processes to which real tar balls would be subjected. Namely, the researchers excluded sedimentation effects (e.g., incorporation of sand particles) and did not examine microbial activity, two processes that could possibly aid in the development of an exterior “crust.”

Heaton et al. (1980) showed that simulated tar balls could form around debris that act as nuclei. Their experiments involved agitating heavy fuel oil in seawater that contained various pieces of debris and with a heat lamp to simulate the weathering effects of the sun. Spherical tar balls formed around the objects and grew to 1–2 cm in diameter after 5 days. The authors did not chemically compare their artificial tar balls to actual tar balls, however. Payne (1982) showed evidence that tar balls grow in size due to the aggregation of many small “flakes” of weathered oil, which adhere to one another when agitated by winds and waves. Savage and Ward (1984) successfully created tar balls using four different types of crude oil. The tar balls were created by placing oil in tanks with distilled water and sand. The tanks were then set on a shaking apparatus under a constant light source. After 4 months of weathering and persistent agitation, the tar balls were analyzed. The measured asphaltene, saturate and aromatic content of the laboratory-created tar balls were compared to those of actual tar balls and found to be within similar ranges of several real tar ball samples.

Laboratory experiments have also been conducted for the purpose of replicating water-in-oil emulsions (chocolate mousse). As these emulsions are the precursors of tar balls, knowledge of the factors affecting their formation and stability can help explain conditions under which tar balls are more likely to develop. For example, it has been found that heavier oils with higher viscosities form emulsions more quickly than low-viscosity/low-density oils and that these emulsions are also more stable (Payne 1982). As viscosity is a function of temperature, lower ambient temperatures also are correlated with more stable emulsions. Thingstad and Pengerud (1983) carried out laboratory experiments on the creation of chocolate mousse from Statfjord oil and determined that photoxidation and agitation were the primary processes required to create stable emulsions. The relative amounts of component waxes and asphaltenes are also believed to be important for determining the stability and structure of laboratory-created mousses. Stable mousses generally involve 65–85 % water incorporation (Fingas and Fieldhouse 2009), and the size of the water droplets in the most stable emulsions is typically less than 10 μm in diameter (Payne 1982).

## Distribution and Prevalence

The existing body of research on marine tar residues consists largely of quantitative or qualitative surveys conducted either on the open ocean (pelagic tar) or on various coastlines (beached tar). Methods for surveying pelagic tar include visual observations made on cruises that are more qualitative than quantitative in nature (e.g., keeping a log of the relative amounts of tar that are observed during a cruise) and towing of nets at sea for the collection and measurement of floating tar (e.g., quantitatively measuring the tar collected over a towing transect). Most frequently, a neuston net is used for collection, which is a net with a fine sieve (~300–500 μm) and rectangular opening that is designed to skim the top layer of water. The nets are towed slowly at a distance from the ship’s hull to avoid its wake, and the collected tar is then weighed. Measurements of pelagic tar are typically reported in units of milligrams per square meter. Quantitative methods of measuring beached tar are less consistent. Some studies have followed a method that was standardized by the United Nations Educational, Scientific and Cultural Organization (UNESCO) that specifies that tar balls be collected on 1–2-m wide transects spanning from the high tide mark and the water’s edge (UNESCO 1984). Other studies have combed entire 2D areas instead of 1D transects, while others have not only collected tar from the surface but also searched for buried tar via trenches dug in the sand (Tsouk et al. 1985) or sediment core sampling (Bernabeu et al. 2013). The Shoreline Cleanup Assessment Team (SCAT) methodology (Owens and Sergy 2003) provides a standardized methodology for surveying coastal regions following oil spills in order to aid in cleanup efforts and was used extensively to monitor tar residues during the DWH recovery (OSAT-3 2013; Michel et al. 2013).

With a sole exception of an experimental technique for using aerial photographs to quantify beached tar accumulation (Golik and Rosenberg 1987), beached tar collection is done by hand, and as a result is time and labor intensive. Depending on the method employed, beached tar measurements may be expressed as g/m or g/m^2^, where m (meters) refers to length of coastline and m^2^ (square meters) refers to beach area. The majority of studies involved regular surveys made over a period of time ranging from weeks to years.

Comparing quantitative and qualitative marine tar ball surveys in various regions provides insight to the source of the tars, as well as the mechanisms influencing their distribution and abundance. Due to the numerous quantitative and qualitative surveys that exist, this manuscript does not purport to cite the literature in its entirety, but rather to outline the main contributions that have aided in this understanding. The majority of quantitative tar studies have been carried out in the Atlantic Ocean or on Atlantic coasts. Studies by Horn et al. (1970), Morris (1971), Butler et al. (1973), Sleeter et al. (1976), and Joyce (1998) quantified pelagic tar throughout various parts of the Atlantic, ranging from the North Atlantic to the Caribbean and equatorial regions. Heyerdahl (1971) made qualitative observations of tar pollution that were observed during transatlantic voyages. Some studies of beached tar on the Atlantic coasts include physical descriptions and observations on few (<10) localized beaches (Okera 1974; Saner and Curtis 1974; Debrot et al. 1995; Butler et al. 1998; Gabche et al. 1998; Debrot et al. 1999; Marquez et al. 2013), while other researchers conducted more extensive surveys that spanned large coastal areas (Georges and Oostdam 1983; Jones and Bacon 1990; Asuquo 1991; Corbin et al. 1993; Debrot et al. 2013).

In the Atlantic Ocean and along Atlantic coastlines, winds can be influential in tar deposition. Along the Sierra Leone coastline, large quantities of tar balls were found washed ashore from May to October 1974 due to onshore southwestern monsoonal winds and increased eastward flow of the Guinea current (Okera 1974). Tar balls were typically deposited on the beach during ebb tides in patterns of crescent-shaped aggregations. At successive spring tides, waves transported the tar balls to the supralittoral fringe, leaving the littoral region unpolluted until the next influx. In Golden Beach, FL, Saner and Curtis (1974) found that east and west winds resulted in more tar deposition on the northern end of the beach than on the southern end. There was also a strong correlation between heavy oil deposition and northeast winds. On the northeast coast of Curacao, Debrot et al. (2013) found that beaches had varying levels and temporal patterns of tar influxes as opposed to beaches on the industrial southwest coast. The lowest influx rates occurred during the rainy season, whereas the highest rates corresponded with periods of more easterly longshore winds. These findings were consistent with debris sampling along Curacao pocket beaches, which revealed that accumulated debris was especially numerous on windward beaches on the northeast coast, whereas debris on the southeast coast was one to two orders of magnitude less (Debrot et al. 1999). Similarly, Sleeter et al. (1976) found substantial amounts of tar deposited along windward shorelines of Caribbean beaches.

Currents and circulation patterns also influence the distribution and abundance of tar in the Atlantic Ocean and along its coasts. Joyce (1998) inferred that the distribution of floating tar in the Western North Atlantic and Caribbean Sea was primarily controlled by large-scale surface circulation patterns. On open ocean beaches along south Bermuda, the quantity of beach tar was found to be primarily controlled by the mesoscale circulation of the Sargasso Sea, rather than winds. As upwellings and convergences passed Bermuda, the beaches collected debris from the oceanic currents; when a convergence passed, tar deposition was relatively heavy, whereas when a divergence passed, tar deposition was minimal (Butler et al. 1998). On Trinidad and Tobago beaches, high concentrations of tar were attributed to residues from tanker bilge cleanings, which were transported by the south equatorial current. In the dry season, northwesterly currents and northeasterly winds produced more tar strandings on Trinidad than Tobago. Conversely, during the wet season when currents are more northerly and southeasterly winds prevail, more tar was found on Tobago (Georges and Oostdam 1983). Beach sampling along Jamaica’s coasts revealed that the highest concentrations of tar occurred along the east coast and in Kingston harbor; these high concentrations were transported by prevailing winds and the Caribbean current. Tar along Jamaica’s north coast was minimal, except during an isolated incident; the west and south coasts were unaffected by tar pollution (Jones and Bacon 1990). Sampling among six countries in the Caribbean illustrated that east coast beaches had higher concentrations and more frequent occurrences of tar balls due to the north and south east trade winds, as well as the influence of the Benguela and North Equatorial currents. Tar ball occurrence also reflected localized seasonal variations in the currents, wind, and wave regimes (Corbin et al. 1993). Cordes et al. (1980) and Romero et al. (1981) surveyed pelagic and beached tar, respectively, in Northwestern Florida to investigate the effects of the Ixtoc I oil rig blowout that occurred in the Gulf of Mexico in 1979. It was expected that oil would travel the Gulf Stream and pollute the waters and beaches in Northwestern Florida; however, neither study found evidence of increased tar levels that could be attributable to the Ixtoc I spill during their study time frames. Knap et al. (1980) made comparisons of beached tar at five locations in Bermuda in the Sargasso Sea for 2 years and concluded that there was not a measurable decrease in tar pollution as compared to surveys 6 years earlier.

Pequegnat (1979) conducted pelagic tar sampling over the Texas continental shelf in the Gulf of Mexico and reported average concentrations of tar that were less than amounts that had been found in the Mediterranean and Sagasso Seas during similar surveys. Pequegnat and Jeffrey (1979) sampled the Gulf of Mexico for benthic tar. They collected 74 samples across the Gulf of Mexico using a tool called a benthic skimmer. Benthic tars were found throughout the sampling region but were found more frequently in the Western Gulf, coinciding with the areas that contained the greatest number of natural tar seeps.

Several studies have been carried out to survey the extent and nature of the residual tars in the Gulf of Mexico regions affected by the DWH oil, which include parts of the Mississippi, Alabama, Louisiana, and Florida coasts (OSAT-1 2010; OSAT-2 2011; OSAT-3 2013). The DWH residues included large mats of sedimented oil, sand, and shell that were deposited in the intertidal and subtidal zones and fragile tar balls with high sand content distributed on the shores (Mulabagal et al. 2013; OSAT-1 2010; OSAT-2 2011; OSAT-3 2013). In order to make a distinction between these sedimented tar residues and typical surface-weathered tars, new terminology was defined by which the tar mats were referred to as submerged oil mats (SOMs) and the tar balls were called surface residual balls (SRBs) (OSAT-3 2013). Some SRBs were believed to be broken off pieces of SOMs washed ashore, while others may have formed directly from the erosion of oiled sands (Michel et al. 2013). It was noted SRBs were frequently found intermingled in shell hash piles following storms in particular (Clement et al. 2012). Wang and Roberts (2013) carried out 11 surveys over the Florida panhandle and Alabama coasts over a period of roughly 1.5 years following the start of the DWH spill, focusing on the cross-shore distribution of five categories of oil types: tar balls, tar patties, tar cakes, oil sheets, and stained sand. They found that the oil distribution was controlled by several hydrodynamic and morphological factors, including the incident wave conditions and maximum high-tide waterline, and that oil residues were often most concentrated in the trough landward of the berm crest.

The Indian Ocean and its coasts have also been extensively sampled for tar residues. Pelagic surveys have been conducted in the Arabian Sea (Eagle et al. 1979; Sen Gupta and Kureishy 1981; Sen Gupta et al. 1993), the Red Sea (Hanna 1983), and parts of the Indian Ocean, and South China Seas (Oostdam 1984; Price and Nelson-Smith 1986). Quantitative surveys of beached tar have been conducted for the coasts of the Maldives (Long et al. 2010), Qatari (Al-Madfa et al. 1999), Oman (Burns et al. 1982; Badawy and Alharthy 1991; Badawy et al. 1993; Coles and Al Riyami 1996), India (Dhargalkar et al. 1977), and Kuwait (Shiber 1989). Observations of relatively free floating tar along the South African Coast after a tanker collision indicated that tar balls formed rapidly and traveled long distances due to currents and winds. In areas of slack currents, tar balls were observed for up to 8 months after the spill (Eagle et al. 1979). In the Indian Ocean, higher concentrations of floating tar balls were found during the southwest monsoon (Sen Gupta et al. 1993). Surveys in the Indian Ocean, South China Sea, and South Pacific Ocean indicated prevailing wind regimes were the main factor for seasonal variations on tar strands. In addition, the highest beach concentrations were found in areas of oil production near tanker routes (Oostdam 1984). After monitoring tar balls in the Maldives, Long et al. (2010) concluded that tar balls in this area were transported by winds and currents, whereas surface floating oil was mostly affected by winds. Along the Omani coast, tar balls were attributed to oil pollution by tankers operating offshore, accidental discharges of oil during tanker loading, or operational discharges from passing vessels (Burns et al. 1982; Badawy and Alharthy 1991). Due to high sea and air temperatures in this region, oil is exposed to relatively high rates of evaporation and photo-oxidation, which causes the oil to arrive as heavy petroleum particulate residues (tar balls). The highest concentrations of tar have been mainly found on the windward side of sand cusps and in bands along the high tide marks (Badawy et al. 1993). In addition, Coles and Al Riyami (1996) found that concentrations of beach tar in this region was spatially and temporally variable, with the highest concentrations occurring 2 weeks after an offshore storm, which was believed to have caused tankers to jettison petroleum.

On the west coast of India, Dhargalkar et al. (1977) found that tar ball deposition was heaviest during the monsoon months, due to the onshore component of the longshore current dominating circulation patterns. After the monsoon ceased, circulation patterns reversed and tar ball abundance decreased or ceased completely. Michel et al. (1993) conducted a number of observational dives and sediment surveys off of the Saudi Arabian coast following the Gulf War oil spill in 1991 and noted the presence of scattered tar balls in the subtidal region; subtidal sediment contamination was due to oil attaching to either suspended sediments or intertidal sediment particles. The greatest contamination occurred in the sheltered muddy basins, most likely resulting from sorption into fine-grained muds. A concurrent survey of the intertidal region indicated a correlation between the nearshore geomorphology and persistence of intertidal oil. The areas most impacted were halophyte marsh and algal mat complexes as well as mudflats at the heads of sheltered bays. In addition, many burrows were heavily contaminated with oil depths reaching over 40 cm as a result of the high porous sand (Hayes et al. 1993).

The Mediterranean Sea has been of great interest to researchers as well and was sampled for tar balls as part of Horn et al.’s (1970) towing survey, as well as several other pelagic tar surveys (Zsolnay 1987; Golik et al. 1988; Kornilios et al. 1998) and observational reports (Oren 1970). The beaches of Spain (Shiber 1987), Beirut (Shiber and Barralesrienda 1991), Russia (Nemirovskaya 2011), and Israel (Shekel and Ravid 1977; Golik 1982; Tsouk et al. 1985; Golik and Rosenberg 1987) have also been surveyed for the presence of beached tar. Pollution along Baltic Sea beaches was found to be dependent on the amount of oil spilled, the composition, meteorological changes, and the type of sedimentary rock on the coast (Nemirovskaya 2011). Shekel and Ravid (1977) found that the degree of weathering and environmental effects on tar balls were important indicators in tracing the source of tar balls on the Mediterranean coast of Israel. Along the Israeli coast, tar balls were found to be more abundant on the northern and central parts of the coast, as opposed to the southern parts. This was due to the proximity of the beach to oil shipping lanes or dumping sites in the sea. During storm events, tar balls were pushed onto the back of the beach by wave action. Along cliffed coasts, tar balls were transported by longshore currents until a gap in the cliff was reached (such as an estuary), at which point the tar balls were directed inland by waves where they were buried or dried and broke into smaller pieces that were dispersed by wind (Golik 1982). Tsouk et al. (1985) also found that specific wave breaking processes, overtopping of offshore obstacles, and wave refraction either sped up or slowed down tar ball deposition along Israel’s northern Mediterranean coast.

Studies in the Pacific include several pelagic tar surveys (Wong et al. 1974; Wong et al. 1976; Shaw and Mapes 1979; Day and Shaw 1987). The Pacific was also sampled as part of the survey carried out by Price and Nelson-Smith (1986) that covered parts of the Indian Ocean. Sampling along 35° N in the Pacific Ocean, Wong et al. (1974) found that peak tar concentrations were associated with subtropical waters, whereas lower tar concentrations were associated with subarctic waters. The findings of Shaw and Mapes (1979) confirmed this, as did those of Day and Shaw (1987). Wong et al. (1976) found that tar lumps in the northwest Pacific Ocean resulted from tank washings on tankers traveling from the Middle East to Japan; tar pollutants became entrained in the Kuroshio current and created a contamination plume that extended downstream for 7000 km across the Pacific Ocean. Shaw and Mapes (1979) found that the maximum abundance of tar in the Pacific Ocean was associated with convergent mesoscale and small-scale surface circulation features. Overall, there was a relationship between tar abundance and mesoscale eddies; cyclonic eddies result in surface convergence, whereas anticyclonic eddies produce surface divergence. Similar to the findings of Butler et al. (1998), areas of divergence had minimal tar quantities. Beached tar surveys have been conducted to measure tar balls occurring near Coal Oil Point on the California coast, a well-known region of natural oil seeps (Del Sontro et al. 2007) and along the Oregon coast to monitor effects of the New Carissa spill in 1999 (Owens et al. 2002). Del Sontro et al. (2007) found seasonal trends in total tar accumulation in which summer quantities were an order of magnitude higher than winter quantities; a multiple regression analysis revealed that 34 % of the tar variability was explained by a combination of onshore advection via wind and low swell heights inhibiting slick dispersion. Along the Oregon coast, Owens et al. (2002) found that 48 % of the collected tar balls were not consistent with those from the new Carissa source oils, and therefore were a result of “background oiling,” not associated with specific known events. The authors note that this is an important consideration that must be considered when developing cleanup criteria. The Coal Oil Point seep field and its surrounding area was also the subject of two studies funded by the USGS (Lorenson et al. 2009; Lorenson et al. 2011) that involved extensively sampling and categorizing both beached and pelagic tar samples believed to have originated from natural oil seeps. Tar deposition varied on a seasonal scale, and seepage was influenced by the spring-neap tidal cycle with more deposition occurring during neap tides (Lorenson et al. 2009).

Holdway (1986) performed an ambitious circumnavigational survey of pelagic tars over a 2-year period and noted that the spatial distribution of tar was highest in the North East Atlantic and Mediterranean Sea. The US Coast Guard conducted a wide-scale pelagic tar ball sampling program in the 1970s (Anderson and Shuhy 1979), in which the N. Atlantic, Labrador Sea, Gulf of Mexico, N. Pacific, Bering Sea, and Gulf of Alaska were sampled. The polar regions were found to have the fewest tar balls, while the greatest concentrations were found in the North Atlantic, North Pacific, and Gulf of Mexico. The low levels of tar in the polar regions are likely to be the result of a lack of natural seeps in these regions along with low tanker and petroleum exploration activity, and hence, these regions have not been extensively sampled. A study by Levy (1986) which surveyed the Canadian Arctic for pelagic and benthic tar residues found the region to be void of either.

It is notable that the above-cited survey-type studies refer almost exclusively to either pelagic or beached tar balls. The difficulties in detecting and observing benthic tar balls or tar mats, as well as the common assumption that beached tar is the direct result of pelagic tar balls, likely explain the lack of surveys regarding these tar residue types. In many of the studies reviewed above, there is ambiguity regarding the origin of observed beached tar. While it appears that most researchers have implicitly assumed the tar balls they observe/collect on the shores arrived there as floating particles, it is not clear whether this is always a valid assumption. In most of these studies, there is no evidence of the method of transport or discussion of the possibility of benthic transport. Few studies have reported the density of beached tar or the percent mass of sand and shell they contain, and even when this information is measured, it is not known whether the incorporated particles were assimilated into the tar ball prior to or post onshore deposition. In light of these uncertainties, it is difficult to determine if beached tar arrived on the shore via surficial or benthic transport.

A study illustrating these difficulties is provided by Balkas et al. (1982). The authors attempted to determine differences between pelagic and benthic tar balls by comparing their densities and chemical compositions. The state of weathering of the tar ball samples was estimated using losses of specific hydrocarbons. The researchers were unable to discern a pattern of characteristics distinguishing the floating and sunken tar balls. While in general, the benthic tar balls were denser than pelagic, neither the pelagic nor benthic tar ball densities varied much from that of the ambient seawater. Temperature was hypothesized to be an influencing factor in causing some tar balls to sink, but it is not certain if the small difference between the expansion coefficients is sufficient to provide the necessary decrease in buoyancy for a floating tar ball to completely sink (NAS 2003).

A second study measuring the specific gravity of benthic, beached, and pelagic tar balls provided more intuitive results (Iliffe and Knap 1979). The authors found that the specific gravities of beached and benthic tar balls were similar, while pelagic tar balls were lighter. The pelagic tar balls were also deposited further ashore by waves and thus more likely to remain beached instead of getting washed back out to sea.

Visually, the appearance of pelagic tar balls obtained at sea has been noted to differ from that of tar balls that have spent time onshore or on the sea floor. While pelagic tar balls are more or less spherical, the latter group tends to be irregularly shaped and flattened (Iliffe and Knap 1979). However, unless a tar ball is known to have been collected shortly after deposition on the shore, these visual cues are not of much use in determining the origin of the tar ball.

Another observation that can be made from the survey studies is that the majority of the quantitative tar residue surveys were carried out in the 1980s, perhaps reflecting an increased focus on the petroleum industry and its environmental effects at that time in light of the prevalence of major oil spills that occurred over the previous decade. Tar balls in particular were more ubiquitous in the 1970s when fewer regulations were in effect to mitigate the release of petroleum into the marine environment due to transport by tankers and have been noted to have declined over the past two decades, most likely as the result of greater oversight on petroleum operations provided by conventions such as MARPOL (Smith and Knap 1985; Peters and Siuda 2014). Less interest is reflected in the literature in the 1990s and early 2000s, but recent years have seen a resurgence in tar residue related studies, likely spurred by the DWH blowout in 2010.

While the above-cited surveys are useful for indicating the controlling factors determining tar distribution, in general, there are sampling considerations to be taken into account when interpreting their quantitative results (Eagle et al. 1979; Golik 1982). Temporal sampling resolution is important in marine tar residue studies because ocean dynamics play an important role in the transport of both pelagic and benthic tar residues. For beached tar balls, local wind patterns and storm activity can “clean” or “dirty” a beach in a short amount of time (Gundlach et al. 1981; Smith and Knap 1985; Tsouk et al. 1985). Similarly, the temporal distribution of pelagic tar balls may change as a result of hydrodynamic factors and water temperatures, which can cause floating tar balls to sink or resurface (Golik 1982; Balkas et al. 1982). Spatial resolution poses additional limits on the accuracy of survey-type studies. Beach morphodynamics and local wave patterns can lead to regions on a shore where tar balls tend to accumulate (Asuquo 1991; OSAT-3 2013; Bernabeu et al. 2013). Currents and gyres can create convergence zones where pelagic tar is concentrated (Atwood et al. 1987). These factors can result in sampling biases skewing the calculated average concentrations of tar residues. Further, some studies do not differentiate between the initial tar ball sampling survey on a shore and subsequent collections, ignoring that the baseline value will differ from following measurements which represent accumulation over a shorter period of time (Golik 1982; Debrot et al. 1995). Ideally, a distinction should be made between standing crop versus rate of accumulation to avoid this inconsistency. In summary, due to the spatial and temporal heterogeneity in marine tar residue distribution in addition to the difficulty in observing benthic or buried tar residues, only crude distribution comparisons can be made, and limited information on tar occurrence and distribution obtained from quantitative surveys. Although the SCAT method standardizes sampling techniques to circumvent many sources of inconsistencies, it has been used for cleanup purposes instead of regular monitoring of tar accumulation (Del Sontro et al. 2007).

## Chemical Composition and Tracing

A second class of marine tar residue studies are those in which chemical analyses of the tar residues have been carried out, either independently or in conjunction with quantitative surveys. Typically, these analyses are conducted to determine the origin of the tar residues by comparing the tar composition to that of suspected source oils (e.g., a specific tanker, spill, or natural seep). This is colloquially referred to as “fingerprinting” the tar residues (Ehrhardt and Blumer 1972). In other instances, laboratory analyses have been completed to study weathering (Albaiges and Cuberes 1980; Hegazi et al. 2004), microbial colonization (Itah and Essien 2005), or the presence of potentially harmful pathogens on tar ball surfaces (Tao et al. 2011; Kiruri et al. 2013). Methods used in laboratory tests are numerous. Frequently, a number of techniques are employed concurrently to aid in the characterization of tar in terms of age, degree of weathering, biodegradation, and possible sources. There are two main challenges in developing fingerprinting techniques (Hartman and Hammond 1981). First, correlating fresh source oil samples with the weathered tar samples is difficult because the composition of oils changes as they undergo weathering; therefore, a method must be able to detect similarities in the source oils regardless of the degree of weathering. Second, the method must be sensitive enough to detect the differences, which may be subtle, between source oils. An additional caveat to fingerprinting methods is that initial knowledge of the potential source oils must be available if the goal is to identify a specific origin. Some markers, such as iron content, can give a general indication as to whether the tar originated from a ballast or petroleum storage tank, as iron particles are indicative of the oil having come into contact with rust or other steel equipment (Payne 1982). The presence of paraffin-rich compounds is also indicative of the petroleum industry, ruling out natural seeps as a potential source. Some researchers have hypothesized that paraffins may even aid in the formation of tar balls by acting as nuclei (Blumer et al. 1973). However, for a tar sample to be conclusively linked to a given spill or tar seep, comparison to the suspected source oil must be carried out, which may not always be possible.

Gas chromatography (GC) is a popular method of measuring the chemical composition of oils/tars that was developed by Ehrhardt and Blumer (1972) and involves the separation of a solution into individual components. By analyzing the relative quantities of individual components of a tar sample, comparisons can be made to suspected source oils and a conclusion can be drawn as to whether or not the tar residue originated from those sources. The GC method has been shown to be useful regardless of the changes in composition that occur in tars with weathering. Frequently, several suspected source oils are compared in order to rule out certain sources and indicate a single probable match. GC analyses are further specified by the method of detection, which include thermal conductivity detectors and flame ionizing detection (FID), while spectroscopy methods such as atomic emission spectroscopy (AES) and mass spectroscopy (MS) have been used in conjunction with GC analysis to identify the components that are separated by chromatography. With all of these methods, various extraction techniques are used to first prepare the tar samples for analysis.

Butler and Harris (1975) performed GC analysis and analyzed the paraffin content of a number of pelagic tar balls collected in the Atlantic, as well as provided physical descriptions of all of the samples. The presence of paraffins in all of the samples was indicative of tanker wash sources. Mommessin and Raia (1975) studied 110 pelagic and beached tar samples from a wide range of areas, including the Northwestern Atlantic, Pacific, Sargasso Sea, Florida coast, New York Harbor, and Gulf of Mexico near Galveston, TX. Physical descriptions of the samples were given and chemical analyses performed, including infrared absorbance ratios and volatility measurements (by gas-liquid chromatography (GLC)), in an effort to determine similarities, differences, and possible sources of the various samples. They were able to separate the samples into two types: those from oil waste products that occurred on land and those that had undergone appreciable weathering at sea. Certain carbon components and low sulfur levels were tied to industrial products that were found in the New York Harbor, while high sulfur content such as was found in samples near Florida was indicative of transported crudes in the region.

Wong et al. (1976) tested the paraffin and iron content and performed GC analysis on 94 pelagic tar samples collected in the northwestern Pacific and determined that the high iron content and presence of specific paraffins in the majority of the samples were also indicative of tanker sources. Shekel and Ravid (1977) used GC to determine whether samples of tar balls found on the Mediterranean coast of Israel were composed of weathered crude oil, crude oil sludge, or weathered fuel oil. The degree of weathering was also used to estimate the age of the tar balls, which ranged from 2 weeks to 2 years.

Kennicutt and Brooks (1983) analyzed pelagic tars found in the South Atlantic using a variety of methods, including GC, gravimetry, sulfur content analysis, and fluorescence spectra. They were able to group the tars into two types; more highly-weathered tar believed to represent background values present in the region and fresher tar that occurred in greater volumes and was traced to coastal waters, likely the result of shipping industry pollution. Van Vleet et al. (1984) characterized pelagic tar balls collected from the eastern Gulf of Mexico using a variety of techniques, including GC/MS, GLC, and isotope-ratio mass spectrometry (IR-MS). Their findings indicated that more than half of their tar samples could be attributed to tanker operations, while the rest had unknown sources. Requejo and Boehm (1985) used GC/MS analysis on water column layers in the Sargasso Sea, verifying that the tar originated from a submarine seep on the Venezuelan shelf. Kadam and Rokade (1996) used GC comparison, UV absorbencies, and measured ratios of infrared transmittances to match a tar residue found on Mumbai Beach to a specific tanker that was suspected to be the source. Wang et al. (1998) analyzed tar balls and patties collected on the western coast of North America, spanning from Vancouver Island down to central California, using GC/MS, GC/FID, and carbon isotope measurements. Many of the tar balls were found to be from “Bunker C” type fuel oils (the most viscous, residual fuels for general land and marine use), while the others were unknown. Hegazi et al. (2004) used a combination of GC/FID, GC/AED, and GC/MS analyses to investigate the sources and degree of weathering for six tar balls found along the Alexandrian coast in Egypt. Their analysis was successfully able to assess the weathering extent and identify the origin of the oil. Lucas and MacGregor (2006) used GC/FID analysis to roughly categorize tar/oil types found on a Nova Scotia island, with the goal of determining the cause of oil pollution that was harming the seabird population of the island. By tracing the majority of the samples to crude oil, the authors were able to conclude that oil from tankers was predominately responsible for the pollution; however, they also found that oil collected in the same area and day could have many different sources. GC/MS techniques have also been used to link tar balls to the Prestige oil spill in NW Spain 9 years after its occurrence (Bernabeu et al. 2013).

Other methods of analyzing tar residues using molecular markers include measuring ratios of alkanes, hopanes, and/or polycyclic aromatic hydrocarbons (PAHs) to determine the sources and degree of weathering of the samples. These biomarker methods are frequently employed in conjunction with GC and related analysis techniques and have been shown to be reliable even when significant weathering of the tars has taken place (Wang et al. 1994). Measures of isotopes of sulfur and total sulfur content were used by Hartman and Hammond (1981) to investigate the sources of beached tar on the California coast. They were able to trace the majority of the samples to two natural oil seeps in the region, while a smaller percentage was found to be due to unknown sources. Kvenvolden et al. (1995) used carbon-isotopic and hydrocarbon biomarkers and carbon isotopes to show that several flattened tar balls found on the shores of Prince William Sound, near the site of the Exxon Valdez oil spill in 1989, were actually from unrelated, unknown sources. PAH and alkane comparisons to reference oils were also used by Conde et al. (1996) to determine sources of beached tar balls collected in 1989–1990 on the Canary Islands in the equatorial eastern Atlantic. Using cluster analysis, they found that the majority of their samples could be classified as Iranian crude oil and likely resulted from an Iranian tanker spill off of the Moroccan coast in December 1989. Zakaria et al. (2001) studied tar balls found on the Malaysian peninsula beaches. They analyzed 20 tar balls for PAH content and other biomarkers, tracing many of them to two crude oils commonly shipped in that area, Middle Eastern Crude Oil (MECO) and South East Asian Crude Oil (SEACO). Hostettler et al. (2004) used biomarker and isotope techniques to catalogue a number of tar ball samples off of the California coast. Their main goal was to distinguish between anthropogenic and natural tar balls. Similar to Hartman and Hammond (1981), they found that a large percentage of the tar samples came from the Santa Monica Bay seeps, although in their study, the majority came from seeps near Santa Cruz, while others were found to have undergone long distance transport by ocean currents. The molecular marker method was used by Chandru et al. (2008) to study tar balls that were found on the East Coast of the Malaysian Peninsula, similar to Zakaria et al. (2001), and found that nearly all of their samples were derived from MECO. Peters et al. (2008) took a similar approach, separating 388 Californian tar and oil samples into three “tribes” with similar characteristics, which were broken down into further categories. Lorenson et al. (2009, 2011) used biomarker measurements and isotope ratios of 388 beached and pelagic samples of crude oil, natural seep oils, and tar balls predominately from the Californian coast to develop a predictive model of oil source families to be applied to tars of unknown sources. Suneel et al. (2013a) fingerprinted tar balls beached along the Goa coast using biomarkers, carbon isotope analysis and diagnostic ratios. Comparison to common commercial oils confirmed that the tar balls were formed from tanker spills. They concluded that carbon isotope analysis was particularly useful in determining tar ball sources. Marquez et al. (2013) analyzed tar balls from the Northeastern Venezuelan coast. GC/MS and biomarker analyses showed that the tar balls were likely derived from natural sources, specifically from oil seeps and petroleum originating from the source rocks in the region. Most recently, McKenna et al. (2013) and Mulabagal et al. (2013) applied GC/MS and biomarker fingerprinting techniques, respectively, toward the identification of oils and tars originating from the DWH spill.

Regarding fingerprinting techniques for source identification, it is noteworthy that in the majority of cases, the determination of a match is made by subjectively comparing two or more fingerprints (e.g., chromatograms in the case of GC analysis) without conducting statistical analyses. Exceptions include the fingerprinting studies by Hostettler et al. (2004), Peters et al. (2008), and Rosenbauer et al. (2010, 2011) in which hierarchical cluster analysis (HCA) and principal component analysis (PCA) were employed to find statistically significant similarities between several samples, accounting for many variables including biomarker and constituent ratios.

## Physical Transport

Despite the extensive interest in the source and distribution of marine tar residues, and tar balls in particular, few studies have looked specifically at their transport mechanisms. Several researchers have proposed theoretical transport pathways for pelagic tar residues based on knowledge of surface currents and wind patterns (Hartman and Hammond 1981; Del Sontro et al. 2007; Lorenson et al. 2011); however, few publications include detailed physical or numerical experiments involving tar ball transport, and apparently no studies have been done on the hydrodynamic properties of benthic tar balls as sedimentary particles (e.g., settling velocity, entrainment velocity, and drag coefficient).

### Physical Experiments

An extensive literature review has yielded only two examples of field experiments conducted on tar ball transport. Iliffe and Knap (1979) tagged a number of benthic tar balls in the subtidal zone on a bay in Bermuda and tracked their movement by employing divers to record the locations of the tar balls every 2–3 days. The tar balls were found to move up to 40–50 m from their original locations over a period of 24 days. Greater distances of transport coincided with lower specific gravities and onshore winds, while the direction of transport correlated with the circular currents in the bay. A few of the tar balls migrated on shore at some point during the experiment, but the majority stayed in the subtidal region of the bay, and several others were transported further offshore.

Golik (1982) painted a number of tar balls and released them at several points on shore and in the swash zone (the region of the beach where waves run up and down). Their movement was observed over a period of 5 days. Under the calm seas that occurred over that period, tar balls were found to have migrated up to 43 m longshore. It was expected that longshore transport would be much greater under storm conditions. These findings highlight the necessity to account for lateral transport when conducting quantitative surveys to measure the influx of new tar loads on beaches.

### Numerical Models

Because of the serious environmental ramifications of oil spills, many models have been produced for the purpose of simulating the fate and transport of oil slicks. These models range from hydrodynamically based models that treat the spilled oil as Lagrangian floating particles (e.g., Dietrich et al. 2012) to more complicated models which take into account a number of weathering processes (e.g., Chao et al. 2001). Offshore sedimentation is not generally considered in these oil spill models (Bandara et al. 2011). Other researchers have developed models for the weathering processes alone, such as emulsification (Xie et al. 2007) or evaporation (Fingas 1995). A vast body of research exists on oil spill transport modeling of these types; however, they are not detailed here as the focus of this paper is on tar residues in particular and not the fate and transport of liquid oil. Instead, numerical models that are developed specifically for tar residues are herein reviewed.

Models of tar ball transport, like sampling surveys, are easier to conduct when assuming pelagic tar balls. This is because pelagic tar balls are assumed to be transported as neutrally buoyant particles, and thus, knowledge of specific transport properties of the tars, such as density and entrainment velocity, is not required. Annika et al. (2001) developed a 3D numerical model that simulates the weathering and transport of pelagic oil and includes components for the evaporation, emulsification, beaching, and sedimentation of an oil spill. The model was applied to four test cases in the Greek Seas. The first case served to validate the model by comparing the predicted tar ball transport to observations of tar ball deposition made on the west coast of Crete. Tar ball particles were placed at various points between Crete and Sicily and their trajectories traced over 50 days. The authors determined that the resulting locations of the tar balls were well matched by observations. A second case started with a known oil spill location and time and ran for 6 days. The results gave detailed paths for the oil as it traveled from its initial location; however, no attempt was made to validate the trajectories. A third case involved a continuous source of oil at an underwater pipeline to illustrate the potential use of the model in guiding contingency plans for leaks in submarine pipelines, and a fourth case simulated a potential spill in the Thracian Sea. Overall, the main limitations of the model by Annika et al. (2001) are that the validation was only qualitative in nature, and the assumption of pelagic transport ignores the possibility of benthic tar ball transport or the cycling of tar balls between the beach and the intertidal and subtidal regions.

Suneel et al. (2013b) simulated the transport of pelagic tar balls using a 2D particle tracking hydrodynamic model with meteorological forcing. Their model was developed specifically to investigate the origin of several spates of beached tar balls observed on the Goa coast from 2010 to 2011. It was deduced that these tar balls likely came from tanker emissions since no spills were reported in the area during that period, and the authors suspected that tankers traveling along the international tanker route in particular were responsible for the tar balls. The authors did not discuss their reasons for excluding the possibility of natural sources, but it may be the case that there were no known active oil seeps in that region of the ocean. To test their theory, eight possible scenarios were explored as origin points for the tar balls in the Arabian Sea, including several along the international oil tanker route. The authors found that the simulated tar ball trajectories led to the Goa coast when the tar ball particles were released at points along the international oil tanker route, supporting their hypothesis. The authors did not discuss the possibility of benthic tar balls and implicitly assumed that the fate of all floating tar balls is deposition on the beach or biodegradation. Their model, like the model by Annika et al. (2001), is only weakly qualitatively validated. Similarly, Bacopoulos et al. (2014) used a 2D depth-integrated model to explore the tidal transport of what they assumed were pelagic tar balls off the Atlantic coast in Florida.

The recent work by Dalyander et al. (2014) presents a transport model for benthic tar residues, specifically for remnant tars from the DWH oil spill. They treated the benthic tar balls as sedimentary particles and implemented a number of critical shear stress estimates (i.e., high, low, and medium critical shear stress scenarios) to predict conditions under which the particles would move. While the model is unique in addressing benthic tar transport, it is limited by the lack of experimental data on actual values for the critical shear stresses. The approximations were based on the semi-empirical Shields parameter and extensions of this model which, like most other sediment transport models, were developed for uniformly distributed, spherical particles. The properties of benthic tar balls, which for the DWH event have been estimated to range from approximately rounded to irregular and jagged in shape (Clement et al. 2012), are likely to render their critical shear stress values significantly different from those determined using the Shields model. Moreover, Dalyander et al. (2014) focused largely on along-shore transport, whereas cross-shore processes are likely to be the dominant drivers for the transport and deposition of benthic tar balls under extreme events (e.g., hurricanes and winter storms) that involve strong onshore winds.

## Persistence and Degradation

Three processes contribute to the reduction of oil from the marine environment: combustion, biodegradation, and physical removal. When possible, combustion is often employed as a first step toward containment and recovery of an oil spill. However, combustion is not an option for residual tar, and thus, biodegradation is chiefly responsible for the eventual elimination of tar balls from the environment. This process is hampered by the small surface area to volume ratio of tar balls, which limits the ability of bacteria to break them down (Leahy and Colwell 1990; Atlas 1981). Further, the weathered hydrocarbons in tar contain chemical bonds that are not readily disbanded by microbial action (Atlas 1981). There are few long-term studies measuring elimination of tar residues from the environment, likely because other mechanisms are responsible for hiding the tar from view long before biodegradation concludes. Physical removal of tars can occur by burial, offshore submersion, and manual and mechanical removal. Burial of tar balls on beaches occurs as sediment transported onshore covers beached tar balls (Tsouk et al. 1985; Bernabeu et al. 2013). Manual removal of beached tars is effective but labor intensive. Mechanical methods such as those implemented in the DWH cleanup involve using beach equipment to sieve the sand and filter out tar aggregates. Initial use of these vehicles during the DWH cleanup resulted in the breaking up of the tar residues into smaller pieces that passed through the sifting mechanism (Hayworth and Clement 2011; Owens et al. 2011). However, modifications made to the use of the machines such as slowing the operational speed were effective in reducing this problem, and several field tests during the DWH cleanup resulted in recovery rates of 80–95 % for a single pass (Owens et al. 2011).

### In Situ Studies

Long-term studies that have been carried out after oil spills indicate that tar balls and tar mats can be remarkably persistent in the environment. Vandermeulen and Singh (1994) used fingerprinting techniques to link tar residues on the beaches of Nova Scotia, Canada, to oil spilled in Chedabucto Bay when the *Arrow* ran aground there in 1970, indicating environmental persistence of over 20 years. Small tar mats from the Ixtoc I blowout in 1979 in the Gulf of Mexico can still be found off Mexico shores (Tunnell 2011). Bernabeu et al. (2013) carried out analysis of recurrent tar ball sightings on the coast of Galicia, Spain, more than a decade after the sinking of the *Prestige* oil tanker in November 2002, which resulted in the release of 64,000 t of oil spilled into the North Atlantic. The wreck continued to leak several tons of oil years after sinking. The authors conducted annual monitoring of the affected coastal regions in Northwest Spain, looking for evidence of continued oil contamination. As recent as 2011, collected tar balls in the region have been conclusively traced to *Prestige* oil. Their highly weathered condition suggests that they spent many years in the intertidal zones. Tar balls were also found in extracted sediment cores down to 3.75-m depth, indicating that oil still existed in the environment and was simply buried out of sight. Benthic tar from the *Prestige* spill has also been found to be prevalent along the Galician continental shelf (Serrano et al. 2006).

Owens et al. (1987) performed a unique field experiment on the Arctic Ocean coast of Baffin Island, located in Northern Canada. Their goal was to study the evolution of oil spilled in an arctic climate, and thus, they released 15 m^3^ of crude oil near the coast and made observations on its fate over a 2-year period. After undergoing losses to the atmosphere and ocean, the remaining oil became stranded in the intertidal region. While natural cleaning processes rid the shore of much of the oil over the observation period, an “asphalt” or tar pavement of weathered oil developed on the upper slope of the beach. This type of tar residue has been noted to occur in other spills and to persist for years and even decades (Hayes et al. 1993; Vandermeulen and Singh 1994).

While the above examples demonstrate the lengthy possible residence times of marine tar residues, rates of biodegradation have been shown to be dependent on the source oil (Wang and Fingas 1995) in addition to the specific microbes available and other environmental variables; thus, the persistence of tar from a given oil spill may vary considerably. For example, warmer climates may lead to faster biodegradation, as these environments are more conducive to microbial activity, although the dearth of actual field studies makes this uncertain. Blumer et al. (1973) performed in situ monitoring of two tar residues in two different locations—one on a tidally submerged rock off of the Bermuda coast and a second on a beach in Martha’s Vineyard, MA. They found that the climate differences between the two sample locations had little effect on weathering differences over the first year, although the Bermuda specimen was observed to undergo breaking down of the outer weathered crust after 1 year, while the other sample did not break down during the 13.5-month observation time. The small sample size and relatively short duration of this experiment make it difficult to draw conclusions as to the effects of climate on long-term weathering.

### Laboratory Studies

Laboratory experiments on degradation and weathering were conducted by Albaiges and Cuberes (1980) using crude oil for artificial weathering and tar ball samples to measure various weathering processes. Chemical analyses led the researchers to conclude that biodegradation and sedimentation are likely explanations for the removal of tar residues from the sea surface but offered little insight into how these processes work.

Itah and Essien (2005) measured microorganisms present in/on beached tar balls collected from the Nigerian coast in order to study microbial influences on tar ball degradation. Certain microbes were found to be better utilizers of tar balls as food than others, and thus were more efficient biodegraders of tars. The growth profiles of these various microbes over time were shown to be predictive of the amount of tar ball degradation that occurred.

Investigations on the fate and persistence of the DWH oil are still ongoing and are likely to continue for many years. Samples taken from tar mats (SOMs) located off of the coast of Orange Beach, AL, l8 months after the well was capped, indicated that the oils in the tar mats contained PAH levels near that of the original source oil, indicating the mats had undergone minimal weathering (Hayworth et al. 2011). Mulabagal et al. (2013) performed analyses of tar balls and chocolate mousse collected on Alabama beaches over 2011–2012. They used GC/MS analyses of biomarkers (hopanes and steranes) to determine if the tar balls matched the DWH well oil (MC252) and to estimate the degree of weathering they had undergone. The vast majority of the tar balls they collected were traced to the DWH disaster. These tar balls contained between 76–89 % sand and were described as “fragile, soft, sticky and brownish.” Comparison to mousse collected shortly after the oil was released indicated that the tar balls had not undergone significant weathering during the subsequent 2 years. The qualities of these tar residues indicate that they were likely formed via sediment-mixing instead of surface weathering. A few tar balls were found that did not fit this description and instead were hard, highly weathered, and low in sand content. These tar balls were determined to not have originated from the DWH disaster. The authors felt confident in stating that DWH tar balls can be recognized by visual qualities alone, as virtually none of the tar balls that fit the physical description of DWH tar balls tested negative for DWH oil. They also concluded that the majority of the weathering the tar had undergone was from evaporation and dissolution that had occurred before the tar balls were deposited on the shore.

### Residence Times

Some researchers have attempted to make estimates of the residence time of marine tar residues. Pelagic tars are removed from the sea surface when they sink, disintegrate, or are deposited on shore. Morris (1971) estimated the residence time of a pelagic tar ball as being on the order of 6 months to a year in the Northern Atlantic Ocean, determined as a function of the half-life of tars. Sleeter and Butler (1982) came up with a shorter residence time (1–4 months) based on a mass balance approach comparing estimated standing stock and input rates. However, both of these estimates contain a great deal of uncertainty; the half-life of tar, as measured in a laboratory, undoubtedly varies from that of marine tar residues, which vary so much in their composition and degree of weathering that they likely decompose at differing rates. Similarly, the mass balance approach can only be considered accurate if the standing stock and input rates are accurate, yet only rough estimates of these quantities can be expected. For beached tar, the residence time has been estimated as the time before the tar ball disintegrates, is buried, or is washed back out to sea, noting that in the latter two cases the beached tar may reappear. Hartman and Hammond (1981) used visual observations to deduce that beached tar has a residence time of one to two tidal cycles. Their estimate was based on 24 collection periods (bi-weekly) over a 12-month period on the California coast.

### Discovery and Removal of Tar Residues from the Subsurface

Sunken oil and benthic tar residues present unique challenges to clean up and recovery crews. Methods that are effective for floating oil and tars (containment, burning, aerial reconnaissance, skimmers) are not useful once the oil has sunk or been stranded ashore. As such, locating and removing benthic residual oil and tar requires unique approaches that have not been as widely applied as techniques for surface oil. The following case studies illustrate the varied reconnaissance and recovery efforts made for oil spills that have resulted in the formation of persistent benthic marine tar residues.

The Ixtoc I well blowout commenced in the Gulf of Mexico in June of 1979 and continued for nearly 10 months, leading to the release of over 530,000 m^3^ of oil (Gundlach et al. 1981). Snorkeling missions were employed to assess the amount of benthic oil. By early September 1979, it was estimated that nearly half of the oil was buried while 16 % remained on the nearshore bottom. A tropical storm that passed through southern Texas in mid-September 1979 produced two main outcomes: it removed more than 90 % of the existing beached oil and tar and led to the discovery of 36 tar mats in the nearshore region (Gundlach et al. 1981). The mats were composed largely of sediment and water and less than 10 % oil and were conjectured to have been created by the storm activity itself. Additional surveys made following Hurricane Allen in August 1980 showed that several of the tar mats were still visible, and new mats had been uncovered. Small tar mats are still located in the region as of 2011(Tunnell 2011).

Tar balls attributed to the Ixtoc I blowout were first spotted ashore 2 months after the oil started flowing and continued for months, although no testing of subsequent tar ball sightings were carried out in order to validate their origin (Gundlach et al. 1981). Storm activity was found to influence the distribution of tar balls on the affected shores. Repeated surveys of several beaches during August and September of 1979 indicated that the fine-grained beaches were more easily “cleaned” by storm activity than beaches composed of coarser sand and shell fragment. The mechanism behind this finding is that fine-grained sands discourage the incorporation of large particles, and hence, they are more likely to sit on the sediment surface and be removed by incoming waves. Surveys also indicated a pattern of decreasing tar ball size from the shore break past the first sandbar (Gundlach et al. 1981). Few tar balls were found past the first sandbar. While extensive reporting and monitoring were carried out on the tar balls and mats that resulted from the Ixtoc I blowout, no mention in the Ixtoc I reports is made of efforts to remove the tar mats or tar balls resulting from the spill (Gundlach et al. 1981; Hooper 1981).

An example of a marine tar recovery effort is reported by Burns et al. (1995) regarding the Morris J. Berman barge accident offshore of Puerto Rico in early 1994. The tanker released 2860 m^3^ of heavy grade oil, which was observed to start sinking after less than 24 h, forming large mats on the seabed. Because in this case the oil sank quickly, without coming into contact with the shore or undergoing appreciable weathering, these mats were highly liquid and contained only a few percent sand. Consequently, the sunken oil was found to break free and resurface easily. Divers were employed to vacuum as much oil as possible and manually pick up oil from the seabed, while dredging was used in the nearby bay. In general, dredging is not a preferred choice for recovery of sunken oil, as it is expensive, complicated to implement, and does not necessarily result in large recovery volume (National Research Council 1999).

A second example of a tar mat recovery followed the *Buffalo 292* barge spill in 1996, near Galveston Bay. A concerted effort was put forth to prevent the spill from affecting the nearby barrier islands in the Gulf of Mexico. However, within days of the spill, the thickening oil slick was becoming difficult to contain and remove by conventional first-line defense methods such as skimming and pumping. By the sixth day post-spill, the oil slick had formed tar mats and surficial and partially submerged tar patties (Clark et al. 1997). An experimental method of dealing with these sticky tar residues was proposed, by which a pair of shrimp boats would tow a net between them to capture tar mats and patties. Aerial observations were used to guide the path of the boats to regions where the tar existed. Cleanup efforts continued for several days, until all recoverable tar was removed (Clark et al. 1997).

In the case of the *Buffalo 292* spill, the tar mats and patties were easy to track by aerial reconnaissance, as they were fresh enough that they had not yet been obscured by sand cover. However, it is often the case that tar mats and benthic tar balls in the intertidal or subtidal regions have been buried by sediments (Hooper 1981; Hayworth et al. 2011; OSAT-3 2013; Michel et al. 2013). In these instances, a significant hurdle to recovery of the benthic tar aggregates is the difficulty in identifying where they exist. The nearshore region is a high energy environment, with crashing waves and turbid waters, making it difficult to visually identify submerged tar mats and tar balls or to employ reconnaissance methods such as diver searching and autonomous underwater vehicles (AUVs).

The difficulties in discovering submerged tar residues are highlighted by the cleanup efforts following the DWH spill. Submerged tar mats (SOMs) were initially observed in the months following the initialization of the spill by SCAT surveys of the nearshore regions of the Gulf of Mexico during very low tides and snorkeling missions (OSAT-1 2010). Analysis of the SOMs and DWH tar balls (SRBs) showed that they contained high amounts (70–90 %) of sand and shell and relatively unweathered oil (Hayworth et al. 2011; Michel et al. 2013), indicative of residues that formed due to sinking and sedimentation instead of primarily surface weathering. Due to the high energy environment of the surf zone where the SOMs tended to be found, it was difficult to discover and remove them (OSAT-2 2011). As a result, a significant amount of DWH residual oil remained in the subtidal and intertidal regions following the initial cleanup, leading to frequent reoccurrences of tar ball deposition on the coasts (OSAT-2 2011; OSAT-3 2013). The source of the chronic “reoiling” was believed to be from the SOMs breaking apart under hydrodynamic forces, leading to the repeated transport of SRBs on shore, particularly after heavy storm events (Hayworth et al. 2011; Clement et al. 2012). Further investigations led to the identification of four main pathways that DWH tar residues remobilize and migrate to the Gulf of Mexico coasts (OSAT-3 2013): (1) cross-shore transport of broken off pieces of tar mats (SOMs), (2) cross-shore transport of uncovered tar balls (SRBs) and tar patties, (3) longshore transport of SRBs, and (4) uncovering of previously buried tar residues across tidal zones.

Recent reports indicate that there are still an unknown number of SOMs in the Gulf region. In Pensacola, FL, 450 lb of tar were recovered by the Coast Guard in April 2013 after a 2-week search (Blair 2013). On June 10, 2013, BP posted a press release on its Web site announcing that active cleanup operations would be discontinued by the middle of that month for the impacted areas in Florida, Alabama, Mississippi, and Louisiana, citing the “extraordinary progress” that the Coast Guard and BP had made in restoring the Gulf of Mexico coastline to pre-spill conditions (BP 2013). However, significant quantities of tar continued to be discovered in Louisiana, where active cleanup operations continued through 2014. In late June 2013, a large tar mat was uncovered offshore of Isle Grand Terre, south of New Orleans (Smith 2013). At an estimated 40,000 lb, officials reported that the tar mat was composed of 85 % sand, shells, and water, and 15 % oil (Buskey 2013). Additional intertidal and subtidal tar mats were also uncovered, prompting the Louisiana Department of Wildlife and Fisheries to close fisheries in several areas of the Grand Terre Islands (Louisiana Department of Wildlife and Fisheries 2013).

## Effects of Marine Tar Residues

### Ecological Effects

The environmental effects of weathered, coherent tar residues have been studied far less than those resulting from fresh oil. With fresh, liquid oil, the harmful effects on shore and on marine life are well documented. These effects include physical coating, which can lead to smothering, hypothermia, or drowning of animals that rely on water-resistant coats (e.g., birds and otters), exposure to toxic compounds due to oil dissolution into the water, and inhalation of the toxic fumes that are released as the most volatile compounds evaporate (Peterson et al. 2003). Long-term effects can also be seen as the food sources of some species are reduced due to oil effects, and changes in immune system response of animals after exposure to toxic oil compounds is observed (Barron 2012). Some of these threats are not pertinent to semi-solid, weathered tar residues, which do not smother/coat animals and have lost some of their toxic compounds to evaporation.

Although few studies have looked at the toxicity of weathered oils, in situ and laboratory studies on crude oils have indicated that in general the toxicity is reduced as weathering takes place (Neff et al. 2000; Page et al. 2002; Jonker et al. 2006). One of the difficulties in studying the ecological effects of marine tar residues is that all spills involve different circumstances, and the differing variables make it difficult to draw conclusions that can be applied to all scenarios in general. Some of the differentiating factors in ecotoxicity are the type of oil that was spilled, the various rates of the weathering processes (which are affected by the environment, habitat, and climate, for example), and the beach substrate (Vandermeulen and Singh 1994). A report commissioned to study the effects of the DWH oil spill evaluated the threat of residual oils from the spill on a number of wildlife habitats (OSAT-2 2011). A team of several experts, including ecologists, environmental toxicologists, chemists, and scientists measured weathered oil samples from the spill a year later and determined that the majority of the original PAHs in the oil had been depleted. They also used results from oil weathering models to predict the timeline for the depletion of the remaining PAHs and determined that risk to any of the considered wildlife groups from the tars was low. However, there was found to be a wide range in the toxicity and weathering of the samples. For example, while samples of weathered oil buried within 6 in. of the sand on the shores were found to be more than 86 % depleted of total PAHs, submerged tar mat samples were found to contain much higher amounts. These differences can be attributed to variations in the conditions leading to weathering; however, studies have also shown that the initial type of oil determines the change in toxicity in response to weathering (Neff et al. 2000). Because of the variations in degree of weathering and the differences in initial oil composition, tar residues do not pose a consistent environmental risk. For example, the ecological risk from a highly weathered tar ball on the beach cannot be equated to that of a recently uncovered, unweathered tar mat, or tar mat fragment.

The impact of benthic tar residues on bottom-dwelling communities is not well understood. Laboratory and in situ experiments conducted by Kalke et al. (1982) on the effect of weathered oil on estuary-dwelling benthos (planktonic larvae) using weathered oil collected from the Ixtoc I blowout showed that while the weathered oil had little appreciable effect on the laboratory communities, the in situ communities were negatively impacted by the weathered oil through decreased biomass and reduced depth of the oxygenated layer, which could reduce subsurface benthic production and alter nutrient regeneration processes.

Wildlife concerns from tar residues focus mainly on pelagic tar and sea turtles. Pelagic tar pieces can be similar in shape to the Sargassum floats that sea turtles feed on, leading to their accidental ingestion (Carr 1987). Neither adult nor hatchling sea turtles feed onshore, so the risk of harm from beached tar balls is low (OSAT-2 2011). While there have been several reports of marine tar ingestion by sea turtles (e.g., Carr 1987; Tomas et al. 2002; Witherington 2002), the effects of ingestion on sea turtle mortality have not been conclusively determined due to a lack of research (Witherington 2002).

One species of starfish was found to exist in increased numbers in regions in which there were high amounts of benthic tar, and several of these specimens were observed to be ingesting tar mat pieces (Pequegnat and Jeffrey 1979). As this species is known to be a selective feeder, the authors believe that this species may be adapted to deriving nutritional benefit from tar residues.

At least one study has found a decrease in molluscan diversity and abundance in regions that were impacted by tar pollution (Nagelkerken and Debrot 1995). This study compared unpolluted beaches to beaches with a large percentage of tar cover, ranging from 17 to 56 % over the entire shore, with portions of the beach “largely cemented into a solid mass” by the tar. In this case, molluscan abundance and species richness would be negatively impacted whether or not the tar was toxic, as the substrate that forms their habitat had been effectively reduced. Another study, by Guidetti et al. (2000), sampled the benthos in the region of the Haven oil spill 9 years after its occurrence in 1991 off the coast of Genoa, Italy. They measured the tar in various layers of the sediment and compared tar abundance to that of the macrobenthos communities within the sediments and found no discernible pattern. While this study suggests that tar residues in the sediments do not negatively impact benthic species, the time lapse between the sinking oil and the sampling could have influenced this finding, as well as the fact that the oil was burned at the surface before sinking, thus removing much of the harmful, volatile components.

### Human Effects

Recently, researchers have raised new concerns regarding human risks of tar ball contact. Tao et al. (2011) conducted laboratory analyses of the total aerobic bacterial counts on a number of tar ball samples obtained from the Gulf of Mexico coast after the DWH event. They found counts were significantly higher in tar balls than in sand and seawater collected at the same location. In addition, *Vibrio vulnificus* (a bacterium that can cause severe illness in humans) numbers were ten times higher in tar balls than in sand and up to 100 times higher than seawater, indicating that tar balls can act as reservoirs for bacteria including human pathogens. It has also been found that DWH tar balls obtained on the coasts of the Gulf of Mexico contain a large number of potentially harmful environmentally persistent free radicals (EPFRs) (Kiruri et al. 2013). The mechanism behind the formation of the EPFRs in the tar balls is believed to be the partial oxidation of iron in the entrained sediment particles. Theoretically, the EPFRs could sicken humans when accidentally ingested or inhaled, but no evidence exists that any person has been sickened by contact with beached tar.

Despite the lack of evidence of significant danger to humans, beached tar pollution is a recognized deterrent for beachgoers and as a result, can negatively impact the tourism industry. People do not like visiting beaches covered in tar, which can stick to feet and belongings and at a minimum serves as an unpleasant visual reminder of environmental pollution, much like seeing litter strewn about at a park. In decades past, when oil pollution was more widespread and tar balls more ubiquitous, commercial products were sold to beachgoers in order to remove tar after a day at the beach (“Tar Away”; Farrington 1985). However, as public perception and standards for pollution have changed, regularly encountering tar balls on the beach is no longer an expected and accepted occurrence. Thus, a substantial motivation in reducing the amount of tar on beaches is to improve the quality of the amenity beaches and to decrease the negative impacts on tourism. Similarly, an additional economic loss is associated with public perception of seafood from impacted regions being tainted. Even when the known ecologically damaging liquid oils have been removed from the ocean after a spill, the reoccurrence of tar balls on the beaches may perpetuate the stigma of those regions in seafood consumers’ minds.

## Conclusions and Recommendations

Research on marine tar residues has been carried out for several decades, originally instigated by the oil and tar pollution caused by the burgeoning petroleum industry. Marine tar residues vary greatly in their composition, appearance, viscosity, and other properties. These characteristics are transient, changing in response to factors such as weathering and sedimentation as tars are exposed over time. Marine tars can be categorized by their location: benthic, pelagic, or beached. Studies on marine tars have ranged from qualitative and quantitative surveys, chemical/fingerprinting analyses, laboratory replication studies, transport modeling, and environmental toxicity effects. Of these types of research, there is a dearth in the latter three categories. Laboratory-controlled experiments on marine tar residues have been restricted to limited attempts at recreating the water-in-oil emulsions that are the precursors to marine tars and studies on recreating tar balls with artificial weathering. No studies have been carried out on the hydrodynamic properties of tar balls, and there is limited knowledge of their density range and the conditions under which they sink and become benthic particles.

Overall, there is a paucity of data on benthic tars. Research on benthic tars has increased in recent years, but long-term studies on their residence times and distribution are lacking, particularly in deeper waters where surveying is difficult. The challenges of directly studying submerged tar residues motivates the development of methods for distinguishing beached tars as having been deposited via pelagic or benthic transport. Such methods could have basis in chemical and weathering analyses that rely on differences between tars that have spent significant time on the seafloor versus those that have remained on the surface. Physical tagging experiments such as were conducted by Iliffe and Knap (1979) and Golik (1982) could provide additional insight into the fate and transport of tar balls and their cycling on and offshore. Currently, little is known about the eventual fate of tars that undergo this cycling and the time scales of the mechanical and biodegrading processes that may aid in their eventual burial or breakdown.

Perhaps as a result of the lack of knowledge on marine tar properties and transport pathways, numerical modeling of transport of marine tars has been conducted mainly assuming pelagic tar balls. A single study on benthic tar ball transport used estimates of transport properties based on methods for uniform, spherical particles.

Recently, an increase in research and observations on marine tar residues has taken place in response to the DWH oil spill in the Gulf of Mexico. The DWH event has led to a greater focus on tar residues that arise primarily due to the sedimentation of sunken oils, in contrast to pelagic tars resulting directly from surface weathering. An unknown amount of residual tar and oil from the DWH disaster remains in the Gulf of Mexico, and recurrent polluting of the beaches may persist as storms and beach morphodynamics uncover subtidal buried tar mats and/or break them apart into pieces that can be washed ashore.

While many studies have been carried out on long-term effects of oil spills on marine and shore life, very few have addressed the toxicity of marine tar residues in particular. Studies of this nature are needed in order to accurately gauge the risks and benefits of cleanup efforts. Although little is known about the long-term ecological effects of tar residues, highly visible, beached tar residues are a recognized nuisance for the tourism industry and likely negatively impact the seafood industry as well. Long-term in situ studies on marine tar ball and tar mat formation mechanisms, degradation times, and transport are recommended in order to better understand these tar residues and to predict their occurrence. Additionally, hydrodynamic transport experiments on benthic tars are recommended in order to aid in the development of physics-based transport models.
